# Expression of *ADAM10*, *12*, *17*, and *28* Genes in Colorectal Cancer

**DOI:** 10.3390/ijms27167441

**Published:** 2026-08-20

**Authors:** Agnieszka Kalita, Magdalena Sikora-Skrabaka, Karolina Gołąbek, Maria Dąbrowska, Joanna Katarzyna Strzelczyk, Dariusz Waniczek, Andrzej Witkoś, Ewa Nowakowska-Zajdel

**Affiliations:** 1 Department of Nutrition-Related Disease Prevention, Department of Metabolic Disease Prevention, Faculty of Public Health in Bytom, Medical University of Silesia in Katowice, 40-055 Katowice, Poland; aga.idzik7@gmail.com; 2 Department of Clinical Oncology, No. 4 Provincial Specialist Hospital, 41-902 Bytom, Poland; magda_sikora19@wp.pl (M.S.-S.);; 3 Department of Medical and Molecular Biology, Faculty of Medical Sciences in Zabrze, Medical University of Silesia in Katowice, 19 Jordana St., 41-808 Zabrze, Poland; 4 Department of Oncological Surgery, Faculty of Medical Sciences in Zabrze, Medical University of Silesia, 41-514 Katowice, Poland

**Keywords:** colorectal cancer, *ADAM10*, *ADAM12*, *ADAM17*, *ADAM28*, gene expression

## Abstract

The role of adamalysins (ADAMs) has been widely described in many processes related to carcinogenesis, angiogenesis, inflammation, metastasis, and metabolic disorders. Despite numerous studies, their role in colorectal cancer (CRC) remains unclear. The aim of this study was to evaluate the expression of selected ADAM genes in colorectal cancer tissue and corresponding surgical margins. In addition, for a subgroup of patients, the expression of selected proteins from the ADAM family was assessed. The final study group consisted of 67 patients who underwent elective surgery for colorectal cancer. The relative expression of the *ADAM10*, *12*, *17*, and *28* genes was expressed as relative quantification (RQ) and determined by real-time quantitative PCR (RT-qPCR) in tumor tissue and surgical margins. In addition, for a subgroup of 45 patients, the expression of ADAM10, 12, and 17 proteins was assessed by ELISA. Associations between ADAM expression and clinicopathological parameters were analyzed statistically. *ADAM12* gene expression was significantly higher in tumor than in margin tissue (median RQ: 0.995 vs. 0.251; *p* = 0.003), whereas *ADAM28* RQ was significantly higher in the margin (median RQ: 0.400 vs. 0.204; *p* = 0.021). No significant differences were observed in the expression of the *ADAM10*, *ADAM12*, *ADAM17*, or *ADAM28* genes based on tumor stage, sex, substance use, BMI, or age, except for nominally higher *ADAM12* gene expression in patients over 65 years of age (*p* = 0.033). Among patients under 65 years of age with cardiovascular disease (CVD), *ADAM28* RQ in tumor tissue was significantly higher than in those without CVD (*p* < 0.05). In obese patients with CVD, a markedly increased expression of *ADAM28* in tumor tissue was observed, regardless of age (1.469 vs. 0.132; *p* < 0.005). Significant positive correlations were observed between the *ADAM10* and *ADAM17* RQ, and between the *ADAM10* and *ADAM28* RQ, in both tumor and marginal tissues (all adjusted *p* < 0.01). No significant correlations were found between gene expression and corresponding protein levels for ADAM10, ADAM12, or ADAM17. *ADAM10*, *12*, *17*, and *28* are poor biomarkers for colorectal cancer, but their significance may increase in patients with comorbid metabolic disorders. The lack of correlation between protein expression and gene expression suggests the contribution of post-transcriptional and post-translational regulatory mechanisms, which justifies further research.

## 1. Introduction

Colorectal cancer (CRC) is one of the most common malignancies of the digestive system, and its pathogenesis is still not entirely understood. Molecular alternations known to date related to the development of CRC are: Vascular Endothelial Growth Factor (VEGF), Epidermal Growth Factor (EGF), Fibroblast Growth Factor (FGF), Insulin-Like Growth Factor (IGF); system activation and dysfunction of pathways associated with inflammatory processes include Tumor Necrosis Factor Alpha (TNFα), Intercellular Adhesion Molecule (ICAM-1), and Interleukin-6 (IL-6) [1,2,3,4].

Numerous studies on the pathogenic mechanisms as well as predictive and prognostic factors of CRC are currently being conducted. Among the biomarkers studied, a disintegrin and metalloproteinase (ADAM, adamalysin) protein family emerges as one of the key factors participating in carcinogenesis, metastases formation, and angiogenesis. ADAMs represent a group of transmembrane and secreted metalloproteinases that hold promise as predictive biomarkers or targets for pharmaceuticals. The most well-known ADAMs in gastrointestinal cancers are ADAM8, 9, 12, 17, 29, and 33, and the most important ADAMs in colorectal cancer are ADAM8, 9, 10,12, 15, 17, and 28 [5,6].

More specifically, ADAM10 and 17 were primarily described in diseases associated with inflammation. In previously conducted studies, concentration of ADAM17 protein in both the CRC tumor tissue and surgical margin was higher in patients with cardiovascular diseases and type 2 diabetes in comparison to CRC patients without these diseases [7]. Furthermore, these molecules are widely discussed in relation to the pathogenesis and progression of cardiovascular diseases such as hypertension and atherosclerosis. For example, a deficiency in ADAM17 promotes the development of atherosclerosis via the TNF-2 receptor. Moreover, elevated ADAM17 activity was observed in the vascular endothelium of the coronary arteries, particularly in elderly and obese patients, which was associated with an increased risk of acute cardiovascular events during the follow-up period [8]. These proteins are also extensively described in both the process of carcinogenesis and the development of autoimmune diseases. ADAM10 is closely related to the Notch signaling that plays role in intercellular communication, including the pathogenesis of colorectal cancer [9,10]. Xiang Z. et al. reported that blocking the Wnt pathway via the ADAM10/NOTCH2/TCF7L2 axis inhibits tumor growth and increases sensitivity to chemotherapy [11]. It has been demonstrated that ADAM10 overexpression correlates with a higher risk of metastasis in colorectal cancer; therefore, it may represent a promising therapeutic target [12]. ADAM10 is not only involved in the progression of colorectal cancer via the Notch pathway, but also the EGF pathway. Previous attempts to use non-conformation-specific antibodies targeting ADAM10 have been associated with high toxicity. Mashati P. et al. developed the 1H5 antibody, which selectively blocks ADAM10, thereby inhibiting the progression of colorectal cancer via the Notch, EGF, and Wnt pathways. As the authors point out, the 1H5 antibody could be used in the future as a component of antibody-drug conjugate therapy [13]. On the other hand, ADAM17 influences tumor development by affecting the secretion of pro-inflammatory cytokines and TNF-α [14]. Jiaming Li et al. suggest that ADAM17 may influence the development of colorectal cancer, namely: invasion, proliferation, and migration via the TGF-β/Smad pathway [15]. In addition, the role of exosomal ADAM17 in hematogenous metastasis has been demonstrated by causing vascular leakage [16]. These findings suggest that ADAM17 may serve as a CRC biomarker but also be a potential therapeutic target.

Furthermore, the role of ADAM12 and 28 in tumor formation is connected to the IGF-1-dependent pathway. Numerous current studies highlight that obesity and metabolic disorders associated with the IGF pathway increase the risk of and contribute to the pathogenesis of neoplasms [17]. ADAM12 and 28 are responsible for breaking down the IGF1/IGFBP3 complex, the biologically active factors: IGF1 and IGF2 bind via the IGF1R receptor, stimulating cell proliferation and inhibiting apoptosis [18]. IGF signals that are passed through IGF1R induce the kinase activity of the receptor and activate the phosphoinositide 3-kinase (PI3K), protein kinase B (Akt), mTOR, PI3K/Akt/ forkhead box O (FoxO), and Ras/MAPK/ERK1/2 pathways [19,20], which play a role in cancer growth. The significance of this pathway has been demonstrated in the development of breast, lung, and colorectal cancer.

In addition, by participating in the proteolysis of the CTGF/VEGF complex, ADAM28 affects angiogenesis as well [21]. ADAM12 is a complex, multi-domain protein that regulates cell proliferation and movement. Moreover, it can shed heparin-binding, epidermal growth factor-like growth factor (HB-EGFR) to activate the EGFR signal pathway [22,23], shed delta-like 1 to activate the Notch signal pathway [24], interact with type II receptor to activate the TGF-beta signal pathway [25], interact with b1 integrin to regulate cell migration, and therefore promotes angiogenesis [26].

The aim of this study was to determine the expression of *ADAM10*, *12*, *17*, and *28* genes in tumor tissue and matching surgical margin in patients who underwent surgery for colorectal cancer. Additionally, the expression of the genes under study was analyzed in relation to selected clinical parameters of the patients. To the best of our knowledge, this is one of the few studies simultaneously evaluating the expression of ADAM10, ADAM12, ADAM17, and ADAM28 genes in paired colorectal cancer and surgical margin tissues, while additionally relating gene expression to corresponding protein levels and selected metabolic comorbidities. The study protocol has been approved by the Bioethics Committee of the Medical University of Silesia in Katowice, Poland (number PCN/0022/KB1/42/VI/14/16/18/29/20).

## 2. Results

The final study group consisted of 33 men and 34 women (Table 1). In addition, for 45 patients, the expression levels of the ADAM10, ADAM12, and ADAM17 proteins were obtained from our previous study [7].

Performed analyses revealed that expression of *ADAM12* genes is higher in the tumor tissue than the margin, and statistical significance was demonstrated (0.995 vs. 0.251; *p* < 0.01). On the contrary, the expression of *ADAM10*, *17* and *28* was higher in the margin, and statistical significance was demonstrated only for *ADAM28* (0.204 vs. 0.4; *p* < 0.05). These results are shown in Table 2.

No substantial differences in the expression of *ADAM10*, *12*, *17* and *28* genes were found depending on the clinical and pathomorphological stage of the tumor.

**Figure 1:**
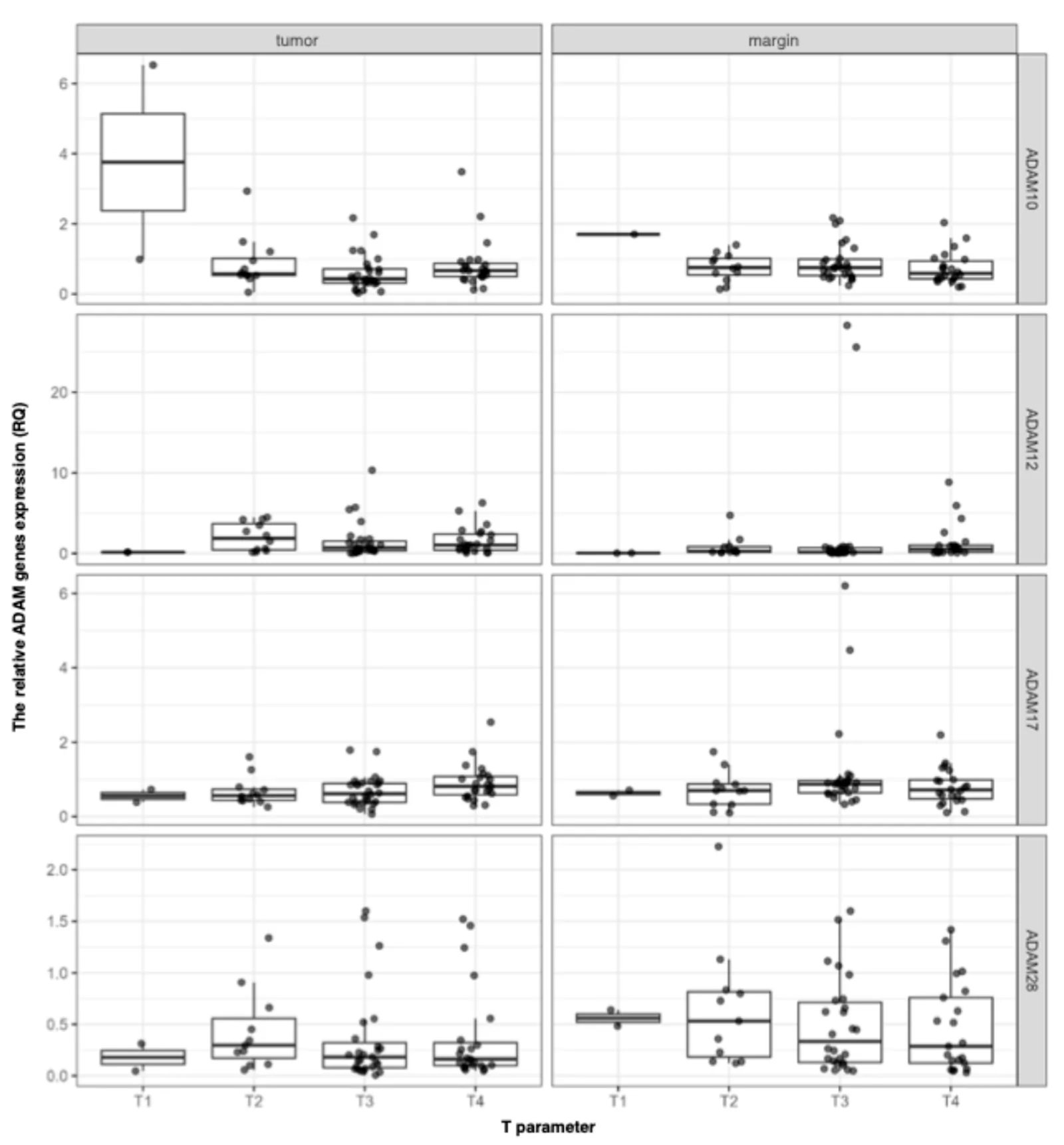
Relative expression (RQ) of *ADAM10*, *ADAM12*, *ADAM17*, and *ADAM28* genes as a function of the T parameter in the tumor and the margin tissue. Although ADAM12 expression showed a nominal decreasing trend with increasing T stage, the association did not remain statistically significant after Benjamini–Hochberg correction.

**Figure 2:**
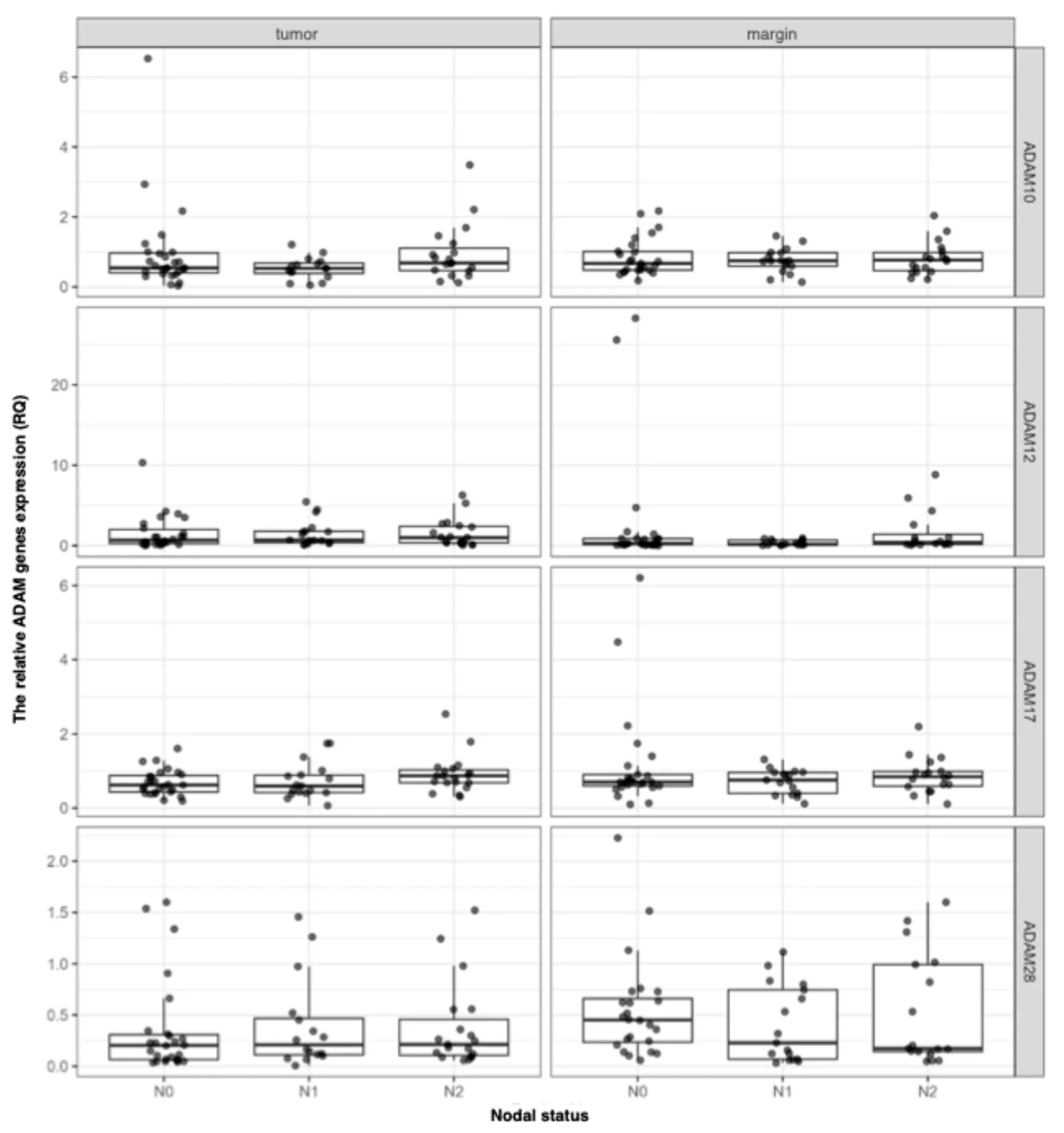
The relative expression (RQ) of *ADAM10*, *ADAM12*, *ADAM17*, and *ADAM28* genes as a function of the nodal status in the tumor and the margin tissue.

The relationship between mRNA expression and T and N parameters was also examined between tumor tissue and the margin. These analyses were conducted because molecular changes in the negative margin—that is, the margin free of tumor cells—could potentially influence the expression of the ADAM genes under study. No correlation was found between the expression levels of adamalysins under study and the T and N parameters, with the exception of *ADAM10* and *ADAM12*. The Kruskal–Wallis test was used to compare expression between groups T1–T4 and N0–2 in tumor and margin tissue. A nominally significant difference between the T groups was observed for *ADAM10* gene expression in the tumor tissue (*p* = 0.0409). However, after applying the Benjamini–Hochberg correction, this result was no longer statistically significant (p.adj = 0.182). In the margin tissue, substantial difference between the T groups was observed for *ADAM12* gene expression (*p* = 0.0456); nevertheless after Benjamini–Hochberg correction, this result also did not reach statistical significance (p.adj = 0.182). The expression of *ADAM12*, *ADAM17*, and *ADAM28* genes did not differ significantly between the T groups. These results should therefore be considered an exploratory trend rather than evidence of a stage-dependent difference. It is worth noting that the small representation (*n* = 3) of the T1 population impacts the ability to unambiguously interpret differences between analyzed groups; therefore, the presented results should be interpreted with caution. These results are presented in Figure 1 and Figure 2.

**Figure 3:**
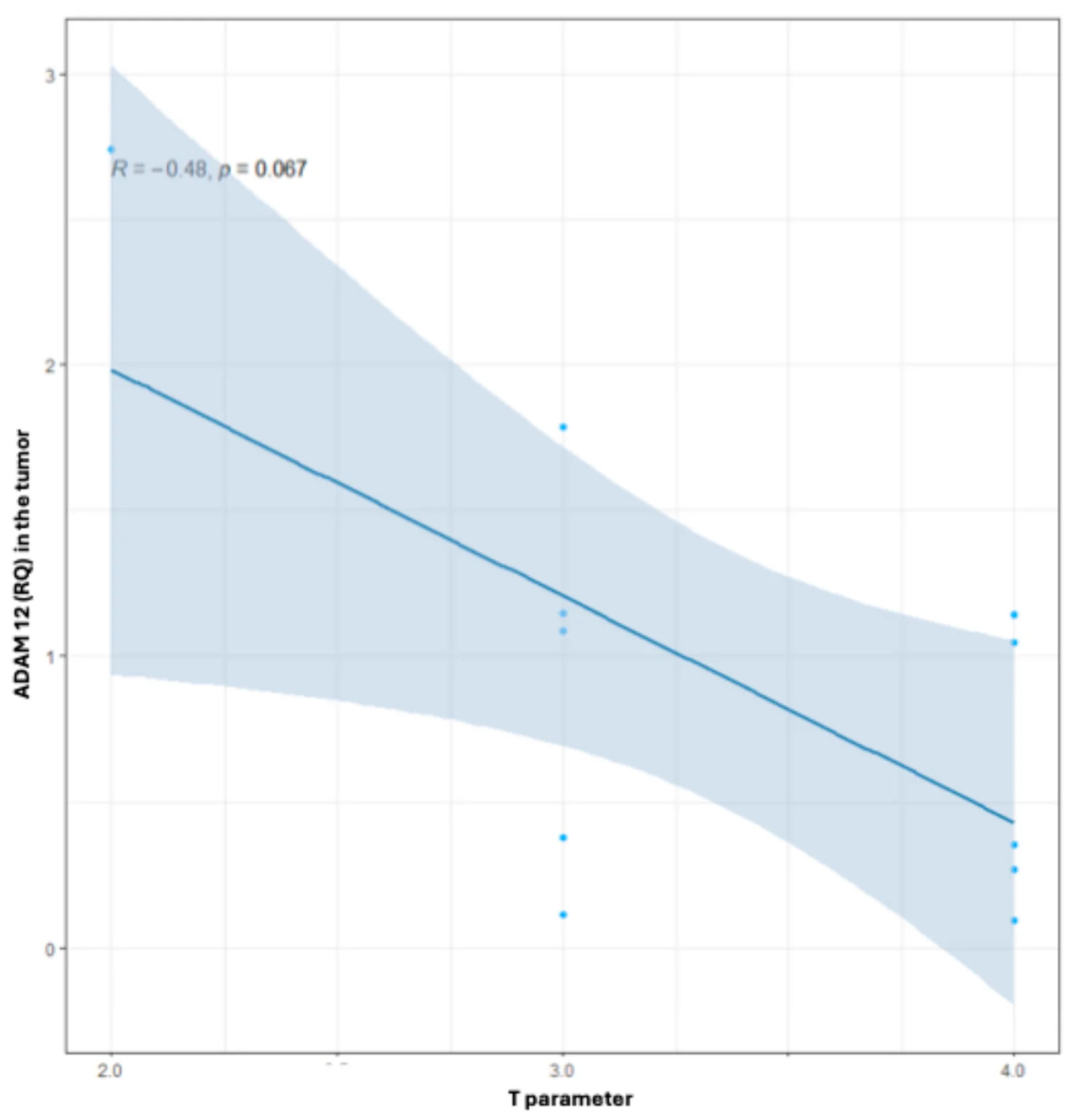
Trend in the correlation between the T parameter and the *ADAM12* gene expression (RQ) in the tumor tissue.

The only moderate negative correlation was observed between the T parameter and *ADAM12* gene expression in the tumor (Tau = −0.48). The value *p* = 0.067 is borderline but does not reach statistical significance (Figure 3).

**Figure 4:**
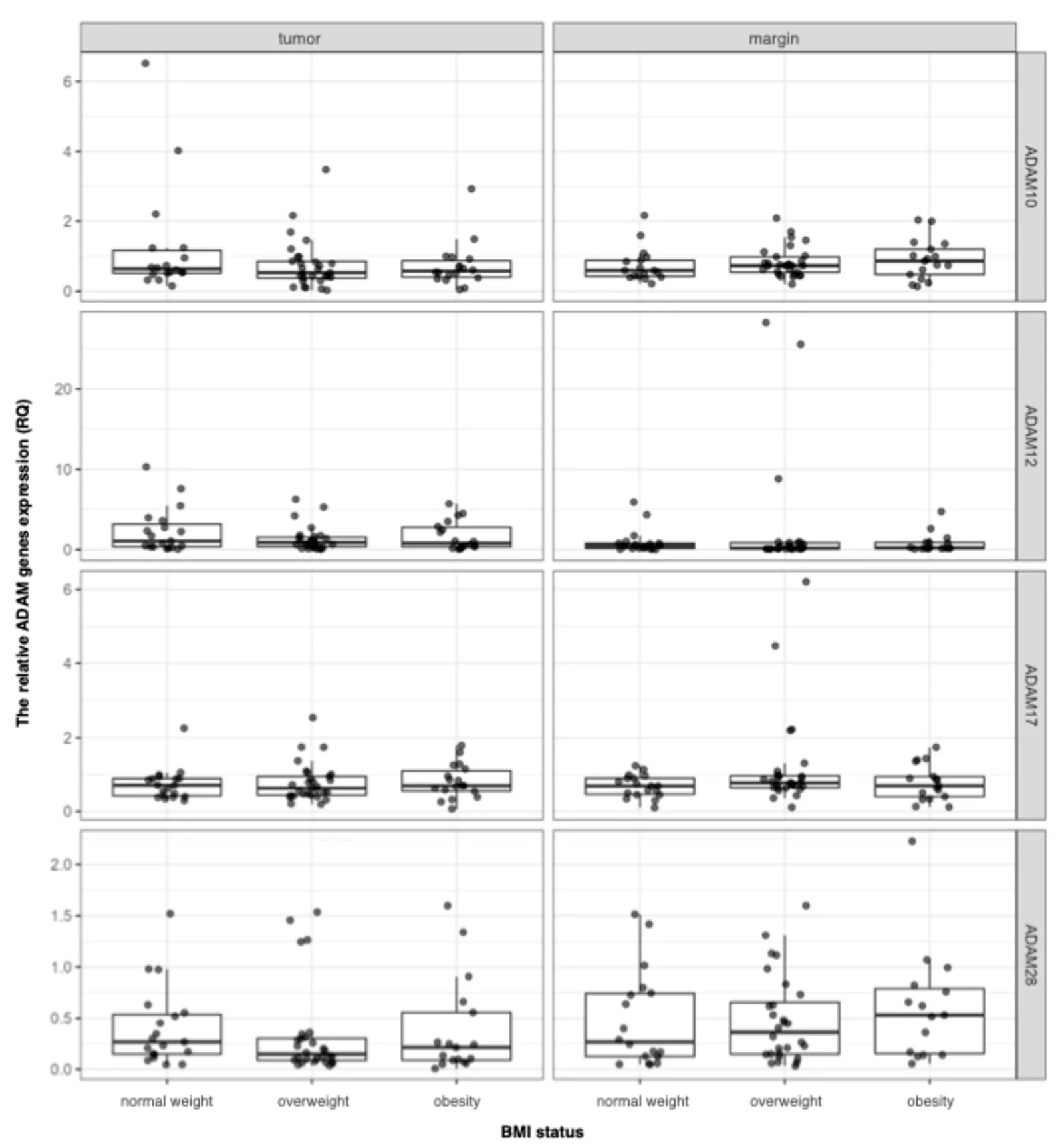
Relative expression (RQ) of *ADAM10*, *ADAM12*, *ADAM17*, and *ADAM28* genes by BMI group in the tumor and margin tissue.

No meaningful differences in the expression of *ADAM10*, *12*, *17*, and *28* genes were found between tumor and margin tissue depending on anthropometric characteristics such as age, sex, or BMI. Furthermore, expression of the analyzed *ADAM* genes in tumor and margin tissue was comparable among patients with normal body weight (BMI 18.5–24.9), those who were overweight (BMI 25–30), and those who were obese (BMI ≥ 30). Figure 4.

In addition, a post hoc Dunn-Sidak analysis was performed for pairwise comparisons of BMI groups. It did not reveal any significant differences between the groups.

In an analysis comparing relative *ADAM* genes expression between patients with normal body weight (*n* = 20) and patients with abnormal body weight (overweight and obese; *n* = 47), no statistically significant differences in the expression of any of the studied *ADAM* genes were found. These results are presented in Table 3.

Only the expression of *ADAM12* gene was statistically higher in the margin tissue in patients over the age of 65 (0.17 vs. 0.53; *p* = 0.033). The expression of *ADAM10*, *12*, *17*, and *28* genes did not differ in patients with nicotine addiction (minimum cigarette consumption defined as 10 pack-years and active smokers or quit smoking less than 15 years ago) or alcohol abuse (defined as a minimum intake of 2.86 g of ethanol per day, which is approximately 2 drinks per week).

In further stages of the analysis, patients were divided into three groups depending on BMI value, and the next division distinguished a group of patients aged 65 and under and over 65 years of age. A healthy body weight was defined as a BMI of 18.5–24.9, overweight as a BMI of 25–29.9, and obesity as a BMI ≥ 30. The expression of *ADAM* genes was studied in these groups of patients with and without cardiovascular disease. Several patients had confirmed type 2 diabetes, but the group was too small to be analyzed.

**Figure 5:**
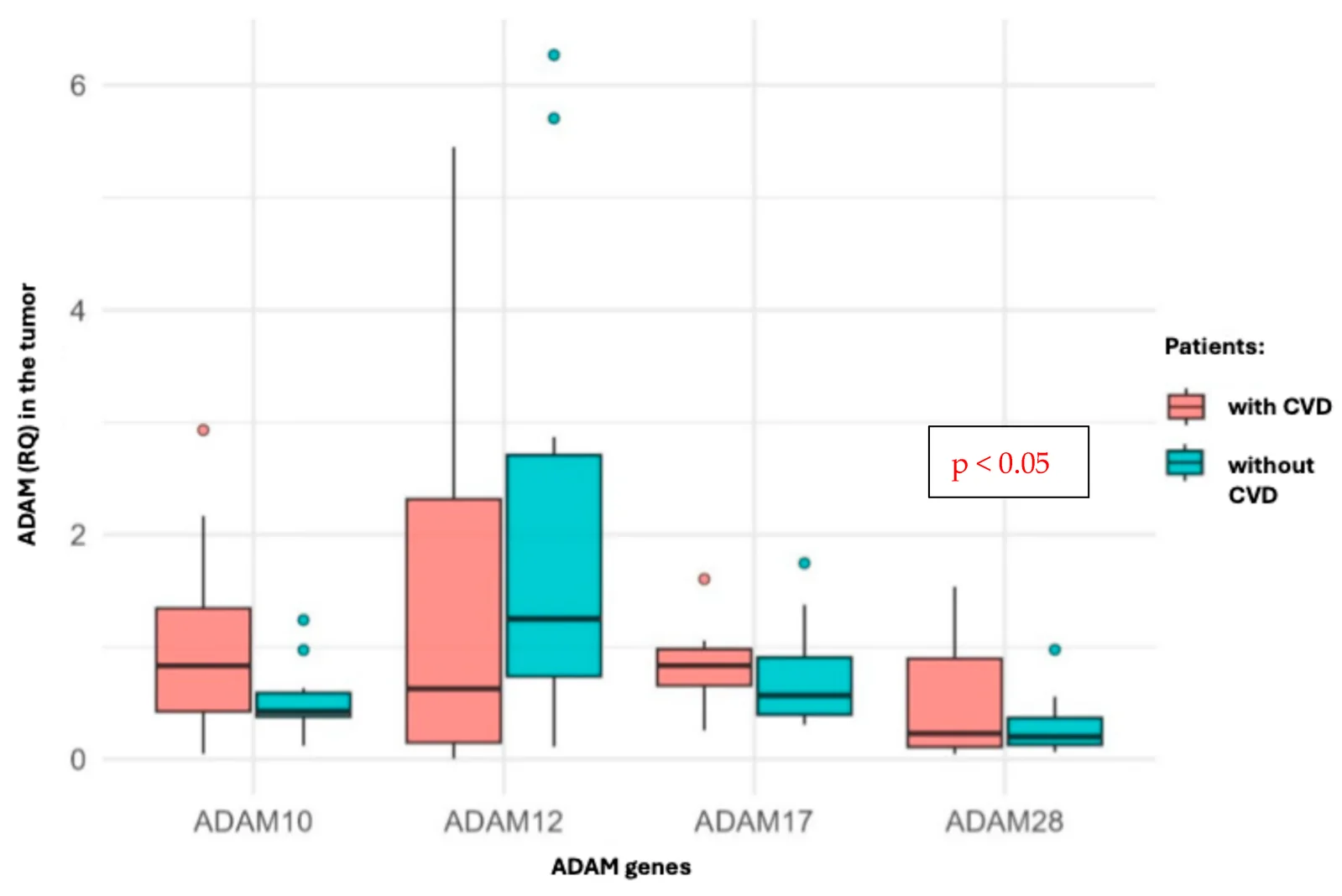
Relative expression (RQ) of *ADAM10*, *ADAM12*, *ADAM17*, and *ADAM28* genes in tumor tissue in patients younger than 65 years according to the presence or absence of cardiovascular disease.

**Figure 6:**
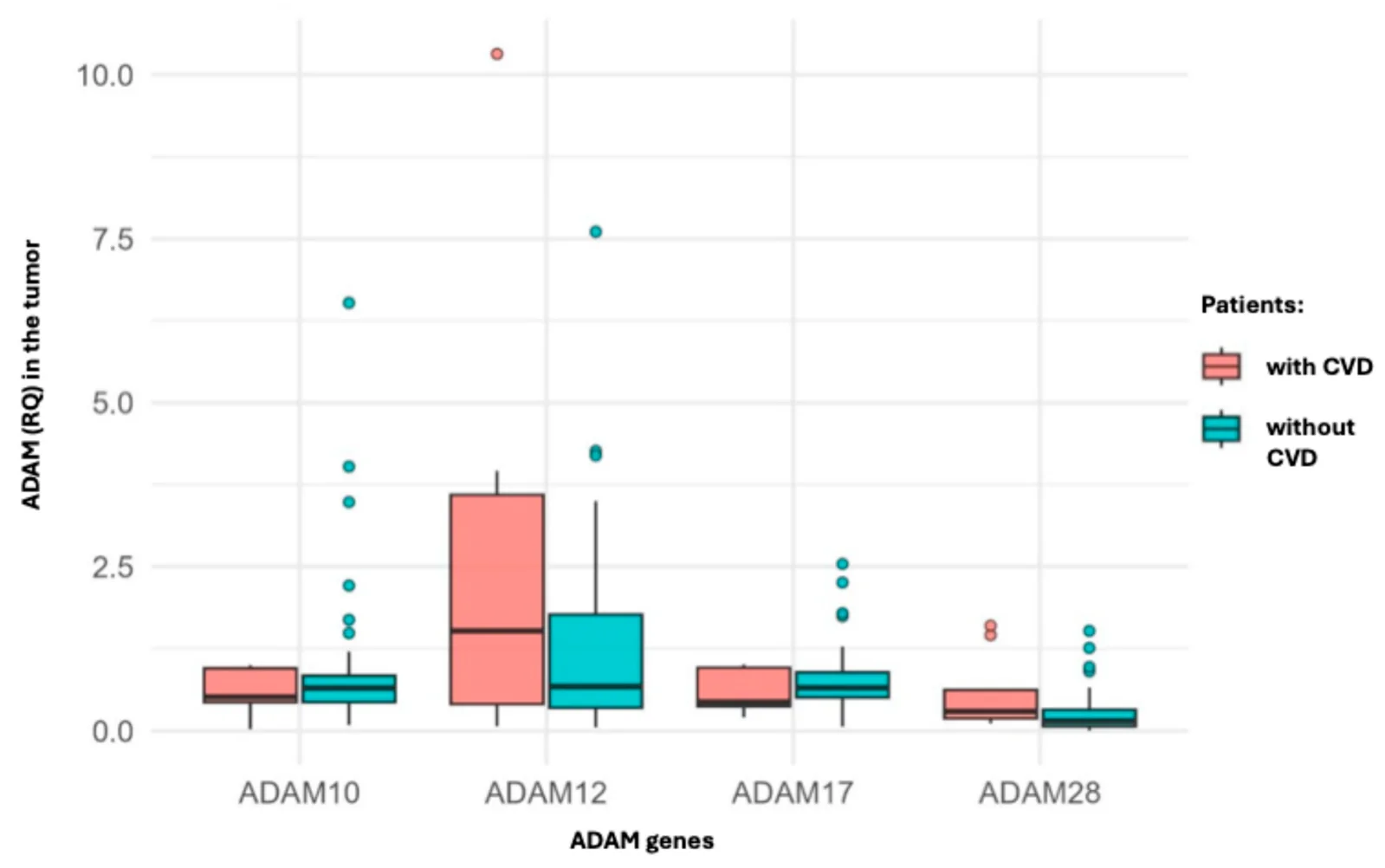
Relative expression (RQ) of *ADAM10*, *ADAM12*, *ADAM17*, and *ADAM28* genes in tumor tissue in patients older than 65 years according to the presence or absence of cardiovascular disease.

**Figure 7:**
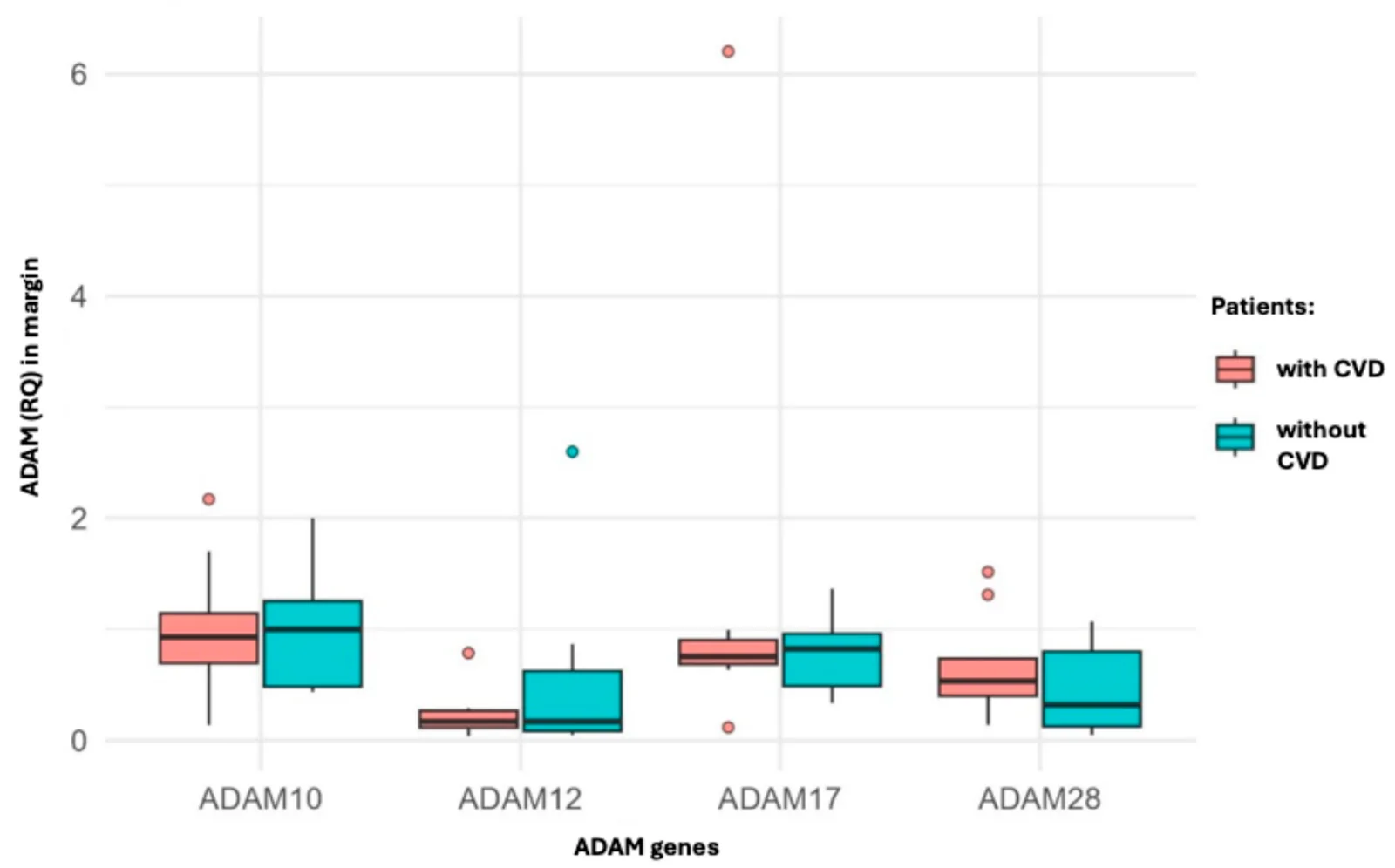
Relative expression (RQ) of *ADAM10*, *ADAM12*, *ADAM17*, and *ADAM28* genes in margin tissue in patients younger than 65 years according to the presence or absence of cardiovascular disease.

**Figure 8:**
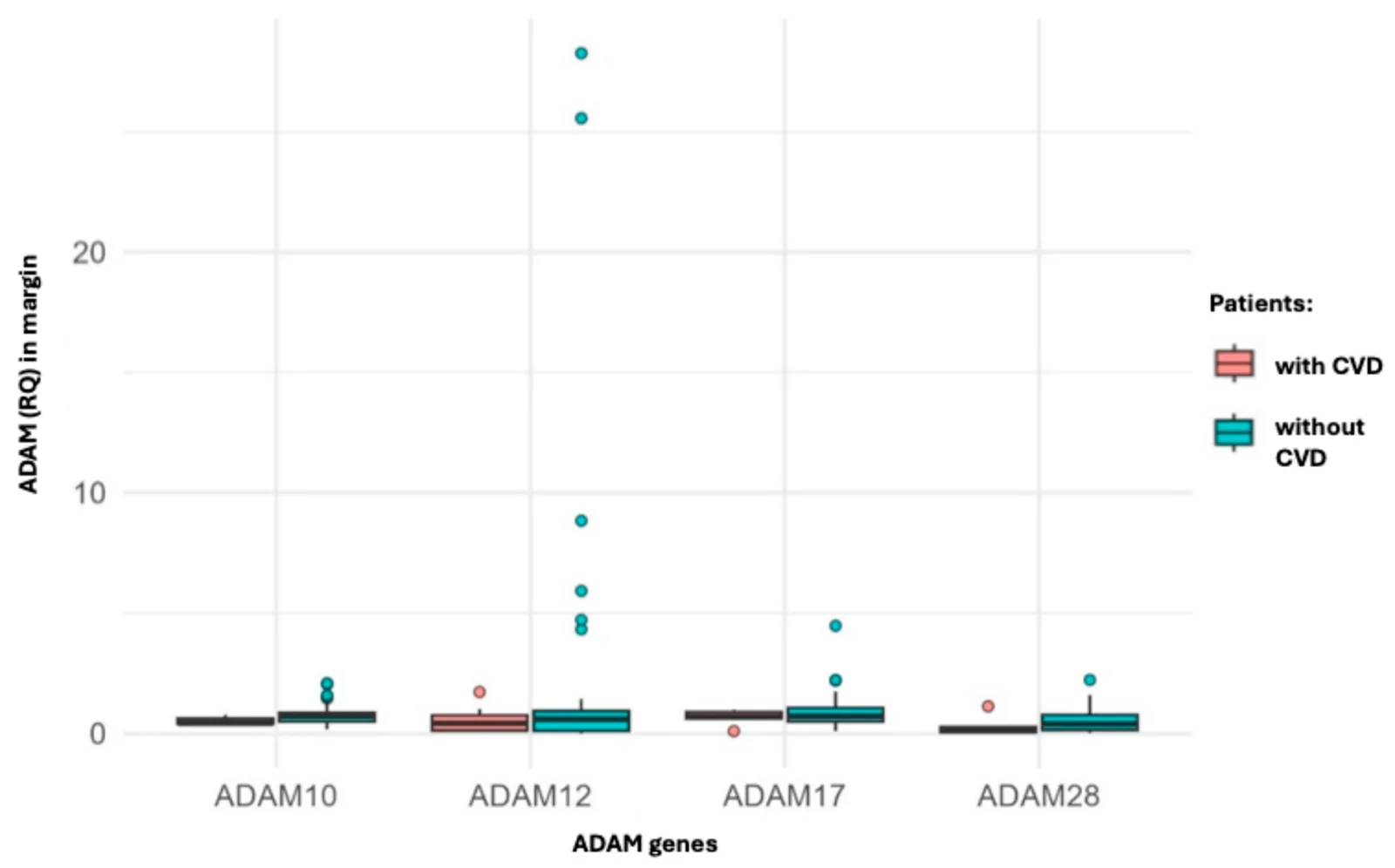
Relative expression (RQ) of *ADAM10*, *ADAM12*, *ADAM17*, and *ADAM28* genes in margin tissue in patients older than 65 years according to the presence or absence of cardiovascular disease.

The relative expression of the *ADAM10*, *17*, and *28* genes in tumor tissue was higher in patients under 65 years of age with cardiovascular disease. But the result was significant only for the *ADAM28* gene (0.299 vs. 0.143; *p* < 0.05). The expression of the *ADAM28* gene was also higher in older patients with CVD, although this difference was not statistically significant. Selected results are shown in Figure 5, Figure 6, Figure 7 and Figure 8.

**Figure 9:**
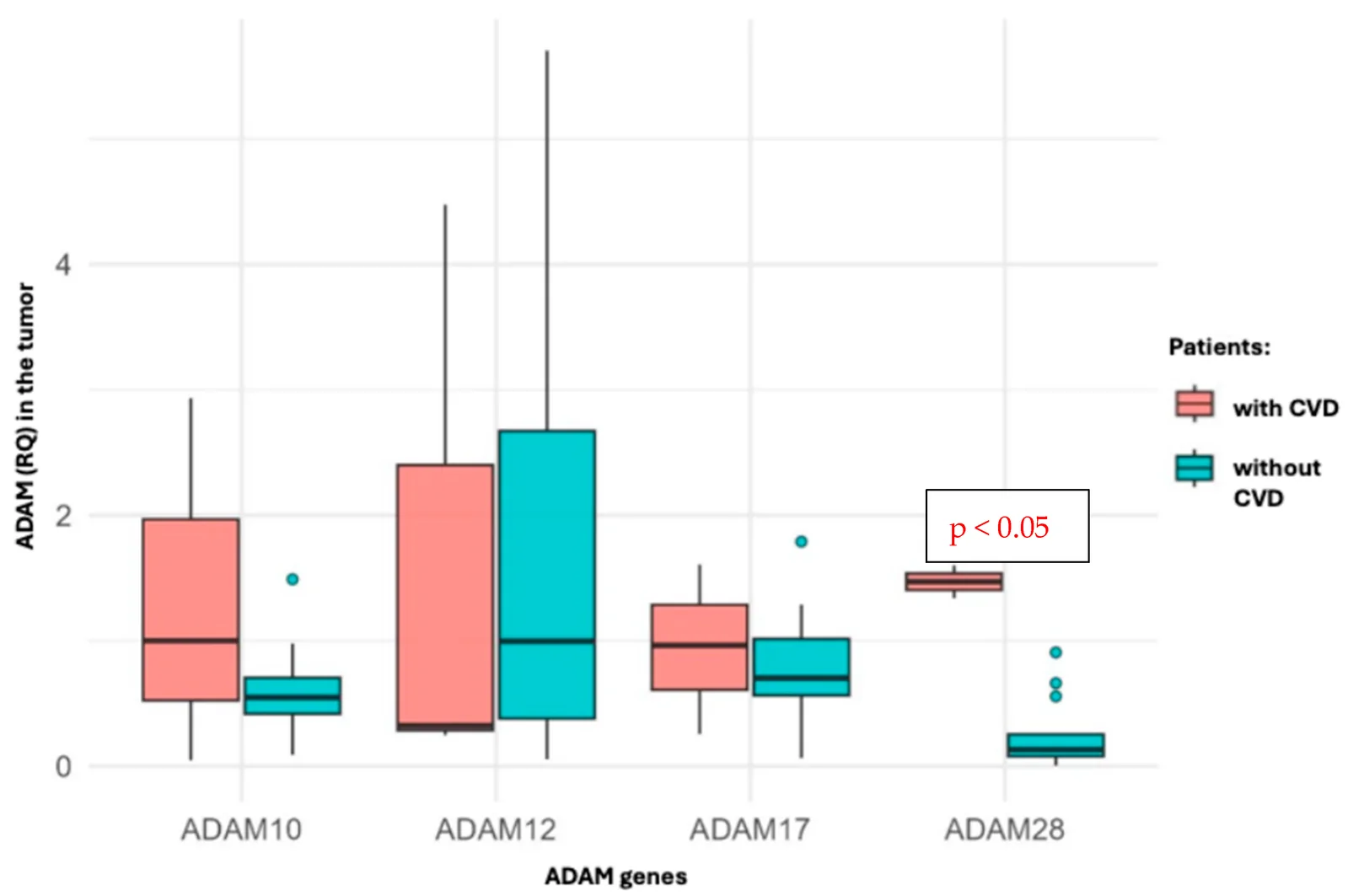
Relative expression (RQ) of *ADAM10*, *ADAM12*, *ADAM17*, and *ADAM28* genes in tumor tissue in obese patients according to the presence or absence of cardiovascular disease.

In patients with obesity (BMI over 30) and concomitant cardiovascular disease, the expression of the *ADAM28* gene in tumor tissue was substantially higher regardless of the patients’ age (1.469 vs. 0.132; *p* < 0.005). This trend was not observed in the margin. Moreover, in patients with normal weight (BMI 18.5–24.9) and who were overweight (BMI 25–29.9) and present cardiovascular disease, the expression of the *ADAM28* gene was higher in the tumor than in the margin, although these values were not statistically significant (Figure 9).

In tumor tissue, major positive correlations were observed between the expression of *ADAM10* and *ADAM17* and between *ADAM10* and *ADAM28* genes. Similar significant positive correlations were found in margin tissue. Moreover, a nominally substantial negative correlation between *ADAM12* and *ADAM28* was observed in the margin, but this result was no longer significant after Benjamini–Hochberg correction. The remaining gene pairs did not present any noteworthy correlations, as seen in Table 4.

**Figure 10:**
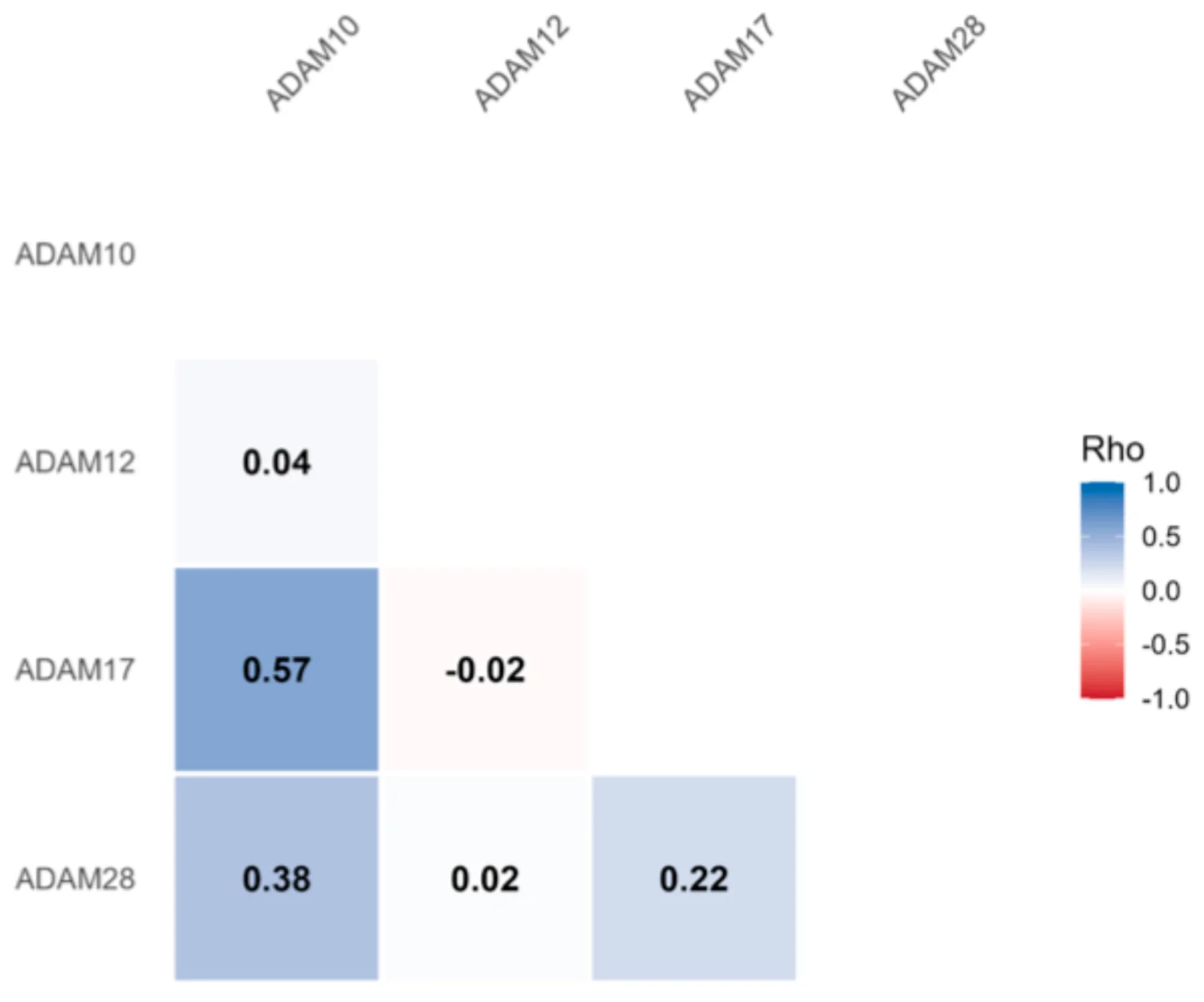
Correlation matrix for the expression of the *ADAM10*, *ADAM12*, *ADAM17*, and *ADAM28* genes in tumor tissue.

**Figure 11:**
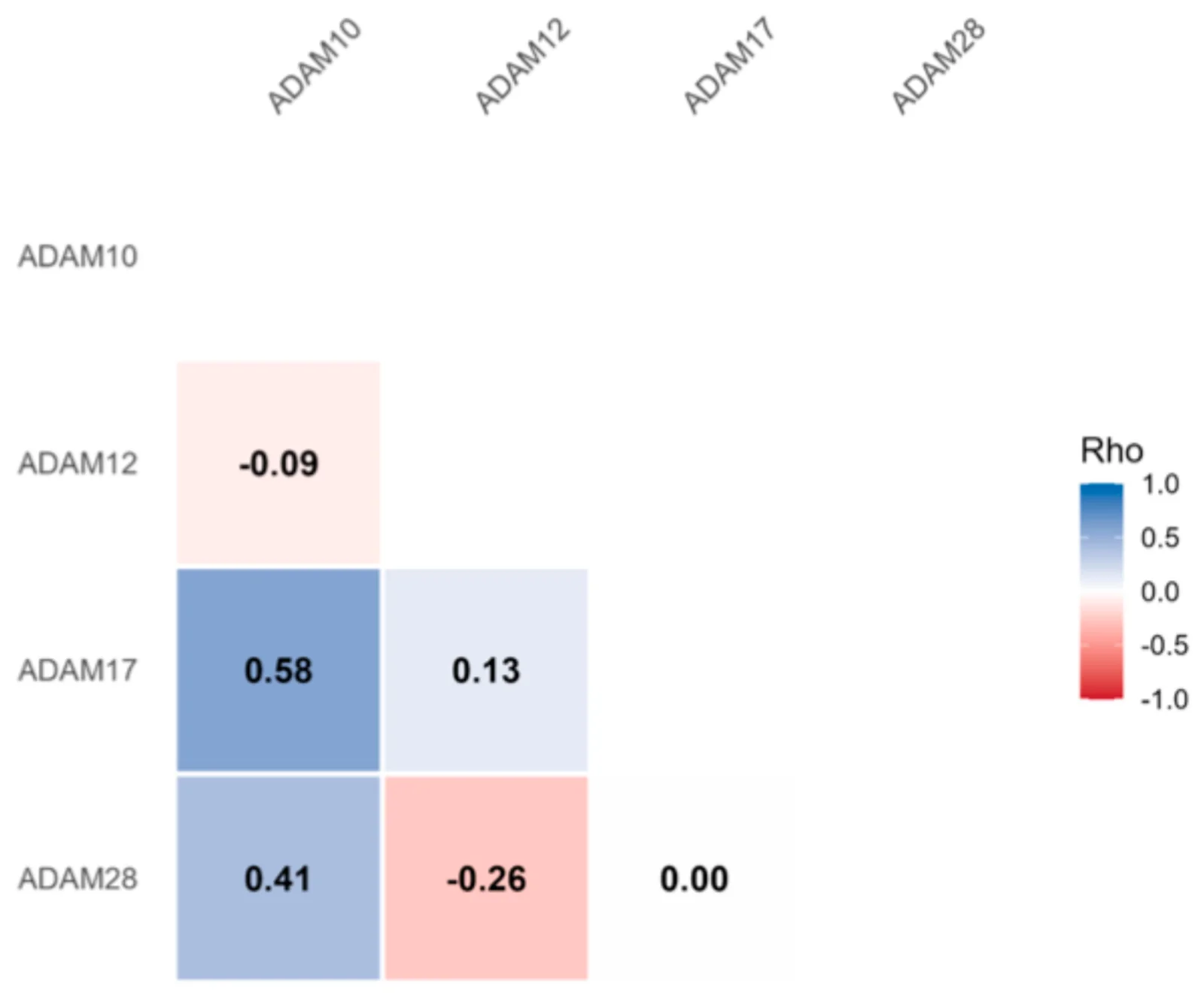
Correlation matrix for the expression of *ADAM10*, *ADAM12*, *ADAM17*, and *ADAM28* genes in margin tissue.

Figure 10 and Figure 11 show the Spearman rank correlation coefficients between the expression of the analyzed *ADAM* genes in tumor tissue and the margin tissue. Color intensity corresponds to Spearman’s correlation coefficient.

**Figure 12:**
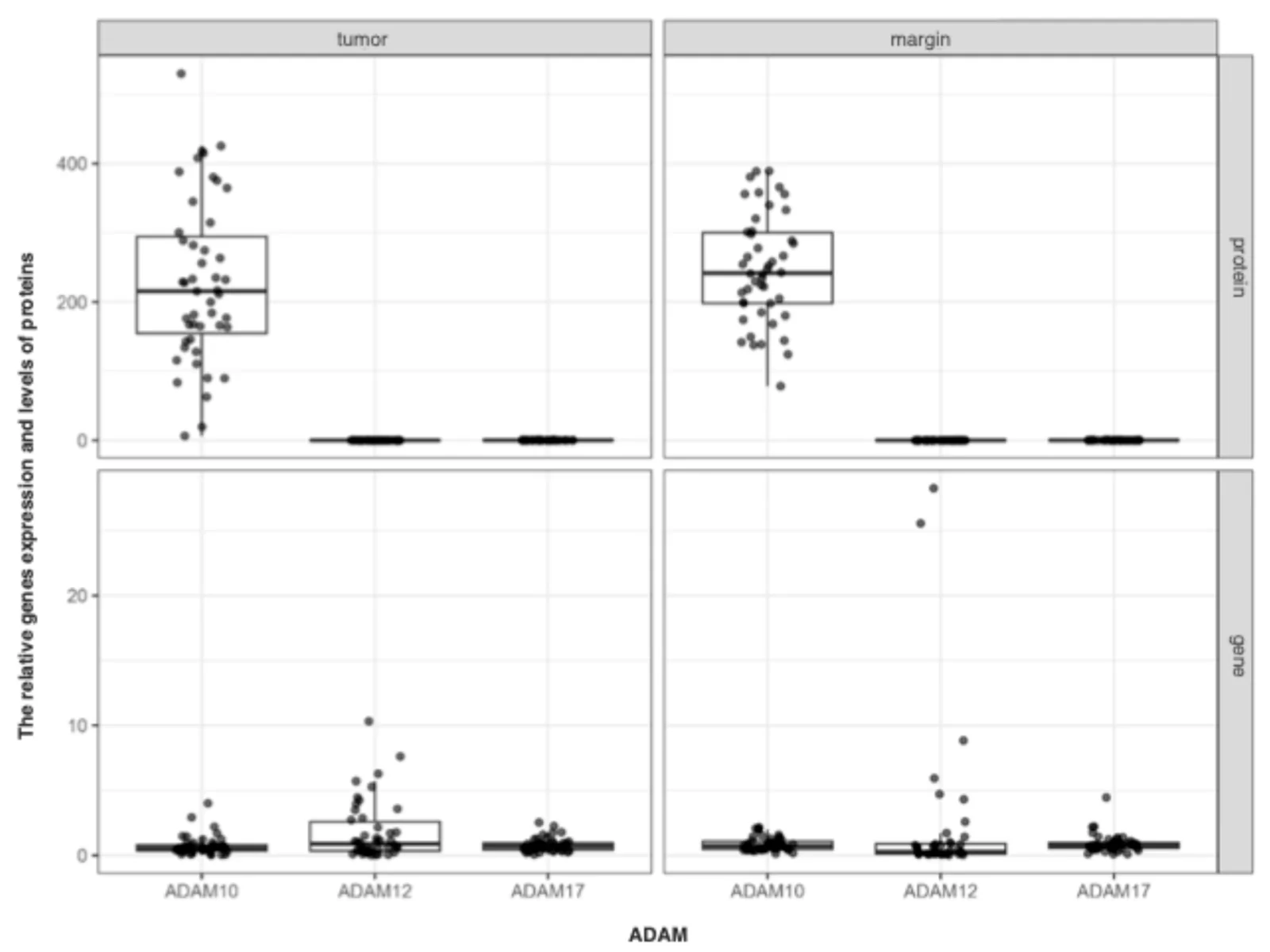
The relative *ADAM10*, *ADAM12*, and *ADAM17* genes expression and levels of the corresponding proteins in a subgroup of patients with protein markers.

Additionally, for the subgroup in which the expression of ADAM10, 12, and 17 protein was detected, we conducted an analysis of the expression of the *ADAM10*, *ADAM12*, and *17* genes and the levels of their corresponding proteins in tumor tissue and the margin, as seen in Figure 12.

**Figure 13:**
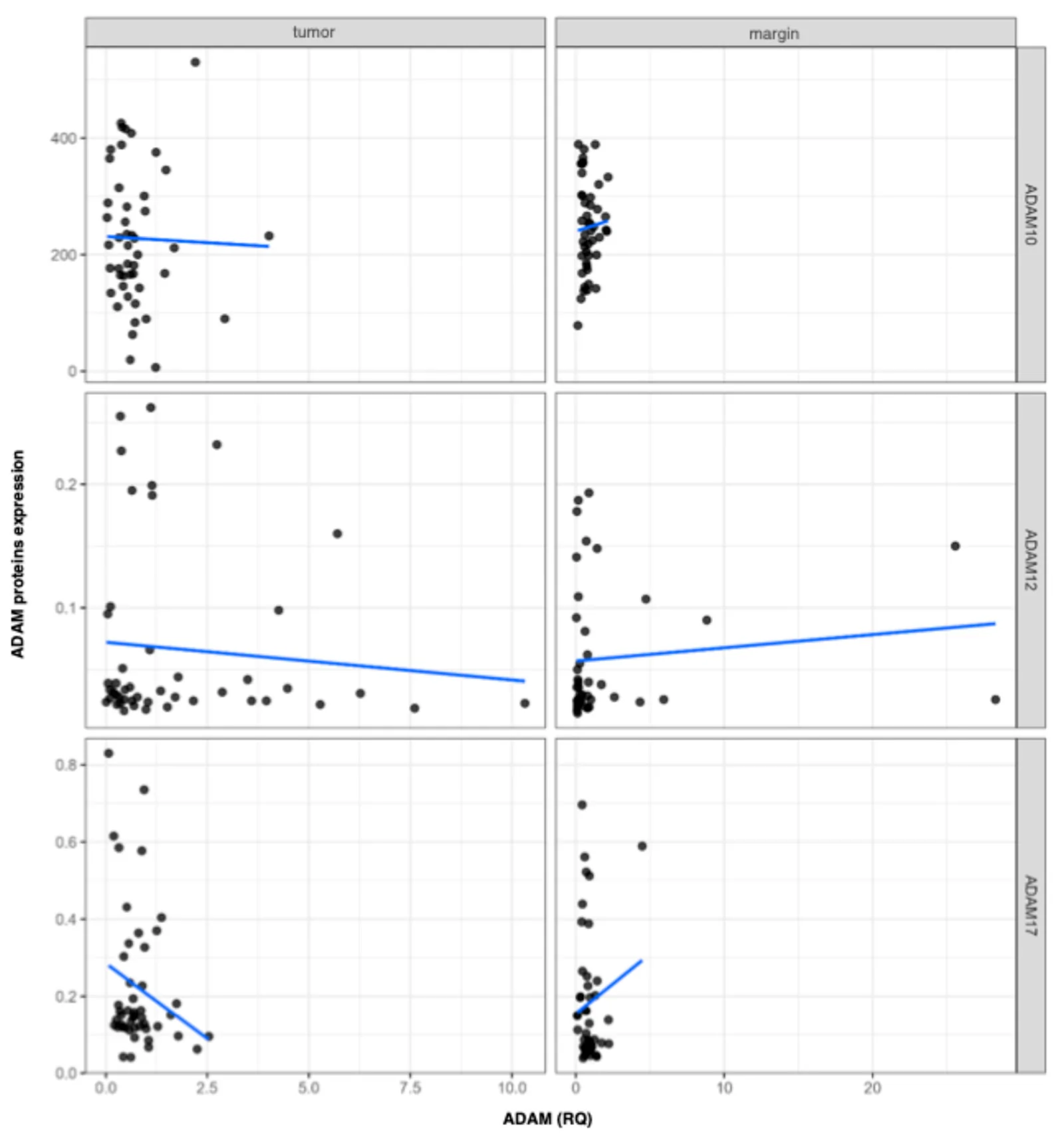
Relationship between *ADAM10*, *12*, and *28* genes relative expression and the levels of the corresponding proteins in the tumor and the margin.

The relationship between gene expression and the level of the corresponding protein was assessed using Spearman’s rank correlation, Figure 13. No statistically significant gene–protein correlations were found for any of the ADAMs analyzed, neither in tumor tissue nor in the margin tissue. 

## 3. Discussion

Based on numerous studies, ADAM 10, 12, 17, and 28 play an essential role in the formation and development of malignant tumors of the gastrointestinal system. However, in most cases, protein expression was studied in tumor tissue or peripheral blood. Data on the expression of ADAM genes in cancer are very limited. This study is a continuation of research conducted at this center. To date, our team has studied the expression of ADAM10, 12, 17, and 28 genes in blood serum and the expression of corresponding proteins in tumor and margin tissue [7,17,27,28]. The results of the studies were unexpected and were not always comparable with those reported by other researchers, but they clearly indicate vital roles of ADAMs in carcinogenesis. Nevertheless, it remains challenging to determine whether they can be considered prognostic or predictive factors as they are involved in various biological processes.

For the pairs of *ADAM10* and *ADAM28* genes, as well as *ADAM10* and *ADAM17* genes, we demonstrated a statistically significant positive correlation in expression levels in tumor and margin tissue. This may suggest coordinated regulation of these metalloproteases independent of tumor status. These correlations are consistent with the results available in publicly accessible databases, such as OncoDB [29]. The clinical significance of gene–gene expression correlations is still being studied [30]. Since the ADAM10–ADAM17 correlation is nearly identical in the tumor and the margin (0.57 vs. 0.58), it can also be assumed that this is a fundamental characteristic of the biology of the tissue under study rather than a tumor-specific marker. The strong association between *ADAM10* and *ADAM17* expression may be biologically relevant as both enzymes are recognized as major sheddases involved in the regulation of Notch [9,10] and EGFR signaling pathways and in the proteolytic release of inflammatory mediators, including TNFα [14,31,32]. Their coordinated expression may therefore reflect activation of shared molecular networks controlling epithelial cell proliferation, differentiation, and inflammatory responses. Since this relationship was observed in both tumor and margin tissues, it is likely to represent a tissue-specific regulatory pattern rather than a phenomenon restricted to cancer development. Additionally, a moderate positive correlation was observed between *ADAM10* and *ADAM28* expression in both tumor and margin tissues, suggesting partial co-regulation of these genes. On the contrary, *ADAM12* expression did not correlate with the expression of the other analyzed ADAM family members, indicating potentially distinct regulatory mechanisms and biological functions.

In our study, *ADAM12* expression in tumor tissue suggested a moderate negative correlation with the T parameter (Tau = −0.48), indicating that higher local tumor advancement may be associated with lower *ADAM12* expression levels. This finding suggests that *ADAM12* may not contribute to tumor growth and local invasion in the study group. In contrast to previous studies reporting increased *ADAM12* [26] expression in advanced CRC and its association with higher TNM stage, deeper tumor invasion, lymph node metastasis, and poor prognosis [33,34], our analysis revealed a moderate inverse trend between *ADAM12* expression and T stage. Although this association did not reach statistical significance (*p* = 0.068), the direction of the observed relationship differs from most published data. This discrepancy may be related to the limited sample size, differences in methodology (mRNA versus protein assessment), or the complex stromal origin of *ADAM12* expression in CRC. Recent studies indicate that *ADAM12* is predominantly expressed by cancer-associated fibroblasts rather than epithelial tumor cells, suggesting that measured transcript levels may reflect variations in tumor microenvironment composition rather than solely tumor cell biology [35,36]. We also found that the expression level of the *ADAM12* gene was statistically higher in the peripheral tissue of patients over 65 years of age, which may suggest that this is an effect related to the tumor microenvironment rather than to the neoplastic process itself.

Our research revealed that expression of the *ADAM12* gene is higher in the tumor tissue than the margin tissue, in contrast to the expression of *ADAM10*, *17*, and *28*, which was higher in the margin. Interestingly, in the subgroup of 45 patients in whom protein expression levels were measured, the concentration of ADAM10 and 12 proteins did not differ significantly between the tumor and the margin. The concentration of ADAM17 protein was significantly higher in tumor tissue (0.148 vs. 0.095; *p* < 0.05). Sikora-Skrabaka et al. found that protein concentration of ADAM17 in the tumor tissue was significantly higher (0.28 vs. 0.2 ng/µg protein, *p* <= 0.01) in patients with concomitant type 2 diabetes, as well as in the margin (0.22 vs. 0.16 ng/µg protein, *p* < 0.05). Moreover, in patients with coexisting CVD protein, concentration of ADAM17 in the margin tissue was substantially higher (0.21 vs. 0.13 ng/µg protein, *p* < 0.01) [7,27]. However, we observed that the expression of the *ADAM17* gene in patients with concomitant CVD is also higher both in the tumor and in the margin, but this result is statistically insignificant.

No significant correlations were observed between the expression of *ADAM10*, *ADAM12*, or *ADAM17* genes and the levels of their corresponding proteins in either tumor tissue or surgical margins.

One of the crucial results of our study demonstrates that *ADAM* gene expression in tumor tissue and in the margin is not identical to protein expression in tumor tissue, and referring to the results of previous studies, also in blood. This indicates that the process leading from the expression of a gene encoding a protein to the start of protein function is very complex and involves several stages, which carry a high risk of error. The lack of significant correlations between *ADAM* gene expression and the corresponding protein levels may be partially explained by post-transcriptional regulatory mechanisms. Buccitelli et al. reviewed studies describing mRNA—protein correlation, the translation mechanism, impact of disruptive factors, and buffering mechanisms [37]. Although mRNA and protein levels usually correlate with each other, Liu Y. et al. concluded that based on gene expression level, we cannot predict and assess protein levels with a high level of certainty. This pathway is still not fully understood [38]. Barett et al. described that one of the reasons for these differences may be the influence of non-coding RNAs, such as microRNAs, on the translation process [39]. MicroRNAs (miRs) are small non-coding RNAs that regulate gene expression by promoting mRNA degradation or inhibiting translation, thereby reducing protein synthesis without necessarily affecting transcript abundance [40]. Consequently, mRNA levels do not always accurately reflect protein concentrations. This phenomenon may be particularly relevant in colorectal cancer, where deregulated miRNA expression has been implicated in tumor initiation, progression, invasion, and metastasis [41]. Furthermore, recent studies have demonstrated that long non-coding RNAs (lncRNAs) and circular RNAs (circRNAs) may act as competing endogenous RNAs, sequestering miRNAs and modifying their regulatory activity [42,43]. Such multilayered post-transcriptional regulation may contribute to the observed discrepancies between the ADAM transcript and protein expression in our cohort and could explain why gene expression data alone were insufficient to predict protein abundance [44]. Our findings are also noteworthy in the context of previous studies investigating ADAM family members in CRC. Walkiewicz et al. showed that in patients with CRC, serum protein levels of ADAM10 and ADAM28 are higher and demonstrated a correlation with the degree of histopathological malignancy and clinical stage in the form of distant metastases [28]. In turn, we found no significant differences in the expression of *ADAM10*, *12*, *17*, and *28* genes depending on the clinical and pathomorphological stage of the tumor. In general, these divergent observations support the concept that gene expression data and protein measurements provide complementary rather than interchangeable information. However, we must consider that the small size of the protein expression subgroup may have limited the statistical power needed to detect weak associations between genes and proteins. Furthermore, analytical variability and differences in the sensitivity of the RT-qPCR and ELISA assays may have further contributed to the lack of significant correlations.

An exciting finding of this study was the significantly higher expression of *ADAM28* in tumor tissue of CRC patients younger than 65 years with concomitant cardiovascular disease. One possible explanation for this phenomenon may be the fact that *ADAM28* plays a role in inflammatory processes and tissue remodeling, both of which impact cardiovascular pathology. Therefore, increased *ADAM28* expression may reflect shared molecular mechanisms associated with chronic inflammation present in both colorectal cancer and cardiovascular disease. The association was observed primarily in patients younger than 65 years, whereas only a non-significant trend was detected in older individuals. This implies that cardiovascular comorbidity influences *ADAM28* expression stronger than age itself.

*ADAM12* and *28* are involved in the IGF-1-dependent pathway [18]. Our research reports overexpression of the *ADAM28* gene in the tumor tissue in patients with concomitant CVD regardless of age, especially in obese patients, that may have potential clinical significance, suggesting that metabolic disorders should be taken into account when studying ADAM family members in CRC. However, given the small size of the subgroups, these results require confirmation in future studies involving large cohorts. Moreover, a preliminary study conducted by Nowakowska-Zajdel E. et al. has evaluated expression of *ADAM28* and *IGFBP-3* in patients with CRC using the microarray technique, and transcriptomic analysis reveals that both *ADAM28* and *IGFBP-3* are differentially expressed when comparing transcriptomes of normal tissue with colorectal cancer from overweight or obese patients [45]. Jowett JB et al. have shown that ADAM28 protein expression level is higher in patients with present parameters of metabolic syndrome like DMT2, obesity, or CVD, which influences the release of TNFα [46]. Furthermore, Herat et al. observed that the expression of *ADAM28* mRNA and protein was significantly elevated in the livers of mice suffering from metabolic syndrome [47]. Our results are consistent with previous studies of protein expression in tissue and blood serum. Unfortunately, since the subgroup of patients with DMT2 in our study was too small, we were unable to isolate it and conduct an analysis.

In summary, our study has once again demonstrated that the selected ADAMs may be rather associated with inflammatory factors in cancer development or may be completely independent of them. Therefore, they cannot serve as standalone biomarkers for cancer diagnosis. However, given the common pathways involved in carcinogenesis and metabolic disorders, further research is needed to identify other factors that influence these pathways. A thorough understanding of described disorders would contribute to the development of new therapeutic targets for lifestyle-related diseases such as DMT2, CVD, and cancer. Further studies are warranted to clarify the role of ADAMs in the pathogenesis of lifestyle-related diseases and to identify additional regulatory factors influencing their expression and activity.

Furthermore, our study is yet another one implying that gene expression does not translate entirely into the concentration of active protein in the tissue. A possible reason for this is that members of the ADAM family undergo complex maturation and activation processes that may weaken the relationship between transcript abundance and protein levels. Therefore, mRNA expression may not directly reflect the amount of biologically active protein present within the tissue.

We are aware that our research has numerous limitations, most importantly the small size of the studied group. This study is a continuation of previous research on the role of ADAMs in CRC. Due to limitations on the amount of tissue material, the subgroup for which protein expression was measured is very limited. The small number of patients in the individual subgroups studied like CVD, age, or BMI limits the statistical power of the tests, so the results should be interpreted with caution. Therefore, it should be emphasized that the results obtained should be considered exploratory and require confirmation in studies involving a larger group of patients. Prior studies focused on protein expression in tumor tissue, surgical margins, and serum. However, the studied groups do not have fully corresponding parameters, so we cannot directly compare the results. Nevertheless, the results obtained confirm the phenomenon of gene and protein expression and point out the need for further research concerning the factors that influence them, such as microRNAs, which will be the focus of our future investigation. Currently, various genes, including the *ADAM* genes, are being extensively studied as potential targets for microRNAs, including *ADAM28* as a potential target for the miR-552 or miR-198. Hebau et al., based on a review of existing research on *ADAM28* in cancer, question the role of *ADAM28* as a biomarker or potential therapeutic target [48].

## 4. Material and Methods

### 4.1. Characteristics of the Study Group

The research group consisted of 72 patients with CRC who underwent surgery in the Department of General and Gastroenterological Surgery with the Oncological Surgery Subdivision in Bytom, Poland. Due to failure to meet the exclusion criteria, 67 patients (33 men, 34 women) were included in the final analysis. The exclusion criteria were patient’s lack of consent to participate in the study, age below 18 years, presence of tumor cells in the surgical margins of the specimen, lack of clinical data in medical history, previous radio-chemotherapy in rectal cancer, confirmed other active or past malignancy (except squamous cell carcinoma of the skin), and diagnosed systemic disease requiring immunosuppressive treatment.

Basic patients’ characteristics, such as anthropometric data, comorbidities, drugs or other stimulants use, imaging, and histopathological results, were obtained from their medical records. The stage of colorectal cancer was determined in accordance with the *TNM Classification of Malignant Tumors*, 8th edition, the Union for International Cancer Control, based on the results of histopathological and imaging examinations [49].

### 4.2. The Tissue Samples

The tissue samples were obtained during routine colorectal resections. The material was immediately immersed in tubes containing RNAlater (Sigma-Aldrich, Carlsbad, CA, USA), a solution used to stabilize tissues and RNA and transported on ice to the Department of Medical and Molecular Biology, Faculty of Medical Sciences in Zabrze, Medical University of Silesia, in Katowice, Poland; there, they were frozen at −80 °C and all assays were performed. The specimen contained a section of tumor (pathologically confirmed adenocarcinoma) and a macroscopically unchanged margin (in accordance with surgical oncology standards) [50]. The absence of cancer cells in the margin was then confirmed histopathologically.

### 4.3. Determination of ADAM Genes Expression

The *ADAM* genes assays have been performed for 72 patients, and the final analysis included 67 patients.

### 4.4. RNA Extraction

The tissue samples were homogenized with FastPrep^®^-24 homogenizer (MP Biomedicals, Irvine, CA, USA) using tubes with ceramic beads for tissue homogenization, Lysing Matrix D (MP Biomedicals, USA), and Buffer RLT (Qiagen GmbH, Hilden, Germany). The RNA was isolated using an RNeasy^®^ Mini Kit (Qiagen, Germany). In addition to the standard procedure, DNase I digestion was performed on the extracted RNA (RNase Free DNase Set, Qiagen, Germany) to remove the residual genomic DNA. The qualitative and quantitative analysis of all isolated RNA was performed by spectrophotometry in a NanoPhotometer Pearl UV/Vis spectrophotometer (Implen, Munich, Germany).

### 4.5. Complementary DNA (cDNA) Synthesis

In the next stage of the investigation, complementary DNA—cDNA was synthesized from the isolated RNA (10 ng in nuclease-free water, EURx Sp. z o.o., Gdańsk, Poland) by the reverse transcription reaction using a set of High-Capacity cDNA Reverse Transcription kits with RNase Inhibitor (Applied Biosystems, Carlsbad, CA, USA) according to the manufacturer’s instructions.

### 4.6. Gene Expression Analysis

The relative gene expression (RQ) analysis was performed by real-time quantitative PCR (RT-qPCR) using specific TaqMan TM Gene Expression Assays (Applied Biosystems, USA). qPCR was performed for 4 genes: ADAM10 (Hs00153853_m1), ADAM12 (Hs01106101_m1), ADAM17 (Hs01041915_m1), and ADAM28 (Hs00248020_m1). The glyceraldehyde-3-phosphate dehydrogenase gene (GAPDH, Hs03929097_g1) was used as an endogenous control. Five surgical margin samples were used as a calibrator. The qPCR for all genes was performed in a volume of 20 µL using 1 µL of cDNA, 10 µL of TaqMan TM Fast Advanced Master Mix (Applied Biosystems, USA), 1 µL of primer and probe mix (TaqMan TM Gene Expression Assays), and 8 µL of H_2_O (EURx, Poland). The thermal cycle for all analyzed genes was 95 °C for 20 s, followed by 40 cycles of 95 °C for 1 s and 60 °C for 20 s. All assays were conducted with a QuantStudio 5 RealTime PCR System (Applied Biosystems, USA). The comparative threshold cycle (Ct) method 2^−∆∆Ct^ was used to determine the RQ.

### 4.7. Determination of ADAM Protein Concentrations

In addition, for 45 of the 72 patients, the concentration of ADAM10, 12, and 17 proteins was determined. The specific methodology is described in the paper by Sikora-Skrabaka et al. [7]. The values were expressed in pg/µg of protein for ADAM10 and in ng/µg of protein for ADAM12 and ADAM17. For this group, analyses of gene and protein relationships were conducted.

### 4.8. Statistical Analysis

The statistical software STATISTICA version 13 (TIBCO Software Inc., Palo Alto, CA, USA) and Microsoft Office Excel 2019 were used to perform all analyses.

Gene and protein expression levels compared between tumor and margin tissue depending on anthropometric characteristics such as age, sex, or BMI were assessed using the Wilcoxon test for paired variables. The normality of the distribution was assessed using the Shapiro–Wilk test. The relationship between T feature and gene expression levels was assessed using Kendall’s Tau correlation coefficient.

Patients were divided into three groups based on body weight (normal weight, overweight, and obese based on BMI values). The second division considered patients’ age (those 65 and under 65 and those over 65). In these groups, the impact of cardiovascular diseases on the studied variables was assessed. The normality of the distribution within the groups was assessed using the Shapiro–Wilk test. The relationships between the variables were assessed using Student’s t-tests for quantitative variables with a distribution close to normal and Wilcoxon tests for quantitative variables with a distribution other than normal, assuming a significance level of *p* < 0.05. In addition, the Kruskal–Wallis test was used for comparisons between more than two groups. Relationships between quantitative variables were assessed using Spearman’s rank correlation. In analyses involving multiple testing, the Benjamini–Hochberg correction was applied. Descriptive statistics are presented as the median, the first and third quartiles (Q1 and Q3), and the interquartile range. A significance level of *p* < 0.05 was used in all analyses.

The results of the analysis are presented graphically using box plots and scatter plots created using RStudio software, version 3. This software is distributed under the terms of the GNU General Public License, either Version 2, June 1991 or Version 3, June 2007. Version 2 can be displayed by RShowDoc(“COPYING”), and Version 3 of the license can be displayed by RShowDoc(“GPL-3”).

## 5. Conclusions

This study demonstrated that the expression patterns of *ADAM10*, *ADAM12*, *ADAM17*, and *ADAM28* in CRC patients are complex and weakly associated with clinicopathological parameters. This set of genes appears to be a poor biomarker for CRC. Nevertheless, their significance may increase in patients with comorbid metabolic disorders. We failed to prove any statistically significant correlations between the expression of *ADAM* family genes and corresponding proteins, either in tumor tissue or in margin tissue, suggesting the importance of post-transcriptional and post-translational regulatory mechanisms. Therefore, we strongly believe that future research should focus on identifying the factors that influence transcription and translation processes or on regulatory factors such as microRNAs to understand the biological role and potential clinical significance of ADAM family members in CRC.

## Figures and Tables

**Table 1:** Characteristics of the study group.

		*n*	*n* (%)
**All**		67	
	Female	34	(51)
	Male	33	(49)
**Age (years)**			
	50–65	29	(43)
	>65	38	(57)
**Clinical stage**			
	I stage	11	(16)
	II stage	12	(18)
	III stage	33	(49)
	IV stage	11	(16)
**Pathomorphological stage**			
	T parameter		
	T1	3	(4)
	T2	12	(18)
	T3	28	(42)
	T4	24	(36)
	Nodal status		
	N0	25	(37)
	N1	19	(29)
	N2	23	(34)
	Metastasis		
	M0	56	(84)
	M1	11	(16)
**BMI (kg/m^2^)**			
	Normal weight	20	(30)
	Overweight	29	(43)
	Obesity	18	(27)
**CVD**		45	(67)
**Stimulants**		17	(25)
	Nicotine	14	(21)
	Alcohol	3	(4)

BMI—body mass index; CVD—cardiovascular disease.

**Table 2:** Relative genes expression (RQ) compared between tumor and margin tissue. (Wilcoxon test for paired variables).

	TUMOR			MARGIN			*p*
	Median	Q1	Q3	Median	Q1	Q3	
***ADAM 10***	0.604	0.402	0.952	0.73	0.479	1.002	0.322
***ADAM 12***	0.995	0.351	2.323	0.251	0.109	0.863	**0.003**
***ADAM 17***	0.695	0.437	0.955	0.752	0.56	0.964	0.939
***ADAM 28***	0.204	0.094	0.382	0.4	0.143	0.748	**0.021**

**Table 3:** Comparison of the relative *ADAM* genes expression between patients with normal (BMI 18.5–24.9) and abnormal body weight (overweight and obese; BMI ≥ 25).

*ADAM* Genes	Patients with BMI 18.5–24.9 (*n* = 20)	Patients with BMI ≥ 25 (*n* = 47)	*p*
*ADAM10* (median [IQR])	0.64 [0.52; 1.16]	0.58 [0.39; 0.90]	0.342
*ADAM 12* (median [IQR])	1.06 [0.35; 3.17]	0.70 [0.32; 1.73]	0.394
*ADAM17* (median [IQR])	0.72 [0.42; 0.90]	0.69 [0.47; 1.00]	0.633
*ADAM28* (median [IQR])	0.27 [0.15; 0.54]	0.17 [0.09; 0.32]	0.222

IQR—interquartile range.

**Table 4:** Correlations between the expression of *ADAM10*, *ADAM12*, *ADAM17*, and *ADAM28* genes in the tumor and the margin tissue.

Tissue	Gene 1	Gene 2	*n*	Rho Spearman	*p*	p.adj BH
tumor	*ADAM10*	*ADAM12*	64	0.04	0.742	0.87
tumor	*ADAM10*	*ADAM17*	65	0.57	0.000	**<0.001**
tumor	*ADAM10*	*ADAM28*	63	0.38	0.000	**<0.001**
tumor	*ADAM12*	*ADAM17*	65	−0.02	0.863	0.87
tumor	*ADAM12*	*ADAM28*	63	0.02	0.870	0.87
tumor	*ADAM17*	*ADAM28*	64	0.22	0.081	0.16
margin	*ADAM10*	*ADAM12*	58	−0.09	0.524	0.62
margin	*ADAM10*	*ADAM17*	62	0.58	0.000	**<0.001**
margin	*ADAM10*	*ADAM28*	60	0.41	0.000	**<0.001**
margin	*ADAM12*	*ADAM17*	59	0.13	0.324	0.48
margin	*ADAM12*	*ADAM28*	57	−0.26	0.051	0.10
margin	*ADAM17*	*ADAM28*	61	0.00	0.980	0.98

*n*—number of observation pairs; Rho Spearman—Spearman’s rank correlation coefficient; *p*—the *p*-value for Spearman’s correlation; p.adj BH—the *p*-value after Benjamini–Hochberg correction.

## Data Availability

The data presented in this study are available on request from the corresponding author. The data are not publicly available due to the protection of patient data.

## Abbreviations

ADAM	A Disintegrin and Metalloproteinase
BMI	Body Mass Index
circRNAs	Circular RNAs
CRC	Colorectal Cancer
CTGF	Connective Tissue Growth Factor
CVD	Cardiovascular Disease
DMT2	Type 2 Diabetes Mellitus
EGF	Epidermal Growth Factor
ELISA	Enzyme-Linked Immunosorbent Assay
FGF	Fibroblast Growth Factor
FoxO	Forkhead Box O
ICAM	Intercellular Adhesion Molecule
IGF	Insulin-Like Growth Factor
IGFBP-3	Insulin-Like Growth Factor-Binding Protein 3
IL-6	Interleukin-6
lncRNAs	Long Non-Coding RNAs
PI3K	Phosphoinositide 3-Kinase
RQ	Relative Quantification
RT-qPCR	Reverse Transcription Quantitative Real-Time Polymerase Chain Reaction
TCF7L2	Transcription Factor 7 Like 2
TGF-α	Transforming Growth Factor Alpha
TNF-α	Tumor Necrosis Factor Alpha
VEGF	Vascular Endothelial Growth Factor
